# Employees’ knowledge and practices on occupational exposure to tuberculosis at specialised tuberculosis hospitals in South Africa

**DOI:** 10.4102/curationis.v43i1.2039

**Published:** 2020-04-01

**Authors:** Lusanda Ndlebe, Maggie Williams, Wilma ten Ham-Baloyi, Danie Venter

**Affiliations:** 1 Department of Nursing Science, Nelson Mandela University, Port Elizabeth, South Africa; 2 Faculty of Health Sciences, Nelson Mandela University, Port Elizabeth, South Africa

**Keywords:** employees, occupational exposure, knowledge, practices, tuberculosis

## Abstract

**Background:**

To prevent the spread of infection of tuberculosis (TB), sufficient knowledge and safe practices regarding occupational exposure are crucial for all employees working in TB hospitals.

**Objectives:**

To explore and describe the knowledge and practices of employees working in three specialised TB hospitals in Nelson Mandela Bay, Eastern Cape, regarding occupational exposure to TB.

**Methods:**

A quantitative, descriptive and contextual study was conducted using convenience sampling to have 181 employees at the three hospitals elected to complete the self-administered questionnaire, which was distributed in December 2016. Three scores on a scale of 0–10 were calculated per participant: *knowledge, personal practice* and *institutional practice*. Descriptive and inferential statistics were utilised.

**Results:**

Approximately, one-third (34%) of the participants were between the ages of 36 and 45 years. Most of the participants (63%) attended high school and less than one-third (28%) had a tertiary qualification. The majority of participants (62%) had not received any clinical training. Participants displayed high scores (> 6) for *knowledge* (75%; mean = 6.65), *personal practice* (68%; mean = 6.12) and *institutional practice* (51%; mean = 6.15). The correlation between *knowledge* and *personal practice* was found to be non-significant (*r* = 0.033). An analysis of variance revealed that Knowledge is significantly related to age and education level.

**Conclusion:**

Employees’ knowledge regarding occupational TB exposure was generally high, but they were not necessarily practicing what they knew. Further research is required regarding appropriate managerial interventions to ensure that employees’ practices improve, which should reduce the risk of occupational TB exposure.

## Introduction

Tuberculosis (TB) is currently regarded as the leading cause of death from infectious diseases. Tuberculosis is ranked ninth as the leading cause from a single infectious agent, ranking above the human immunodeficiency virus (HIV) and the acquired immunodeficiency syndrome (AIDS). The latest World Health Organization global report states that in 2017, there were approximately 10.4 million incident cases of TB alone, and approximately 1.6 million people died from this disease (World Health Organization 2017, 2018). Although the disease burden caused by TB is decreasing globally in most countries, this is not sufficient to obtain the first (2020) milestones of the End TB Strategy as part of the sustainable development goals (SDGs). This strategy entails specific targets for 2030 for a 90% reduction in the absolute number of TB deaths as well as an 80% reduction in TB incidence (new cases per 100 000 population per year), compared with levels in 2015 (World Health Organization 2018).

South Africa is among the top 30 countries with the highest burden of TB (World Health Organization 2018), with an estimated prevalence of 438 000 cases of active TB in 2016. The incidence of TB in South Africa during the past 15 years has increased by 400% (World Health Organization 2017). It is estimated that in South Africa, in a population of approximately 56 million people, 560 000 people (1%) develop active TB disease each year and that 336 000 (60%) of these cases are co-infected with HIV (World Health Organization 2017).

Tuberculosis is an infectious disease caused by the bacteria *Mycobacterium tuberculosis* (Hershkovitz et al. 2015). Transmission of tuberculosis (TB) is by inhalation of droplet nuclei (Iseman 2013). The risk of exposure to TB is higher for employees at healthcare institutions, particularly specialised TB hospitals (Nienhaus et al. 2014; World Health Organization 2017). An evaluation of exposure to TB among employees at a medical centre in the United States revealed that healthcare personnel are at a three times higher risk of occupational exposure to TB than the general population and that there are gaps in the implementation of administrative, engineering and respiratory protection controls (De Perio & Niemeier 2014). Infection of TB can occur when employees and patients come in contact with persons who have unknown TB, and not receiving treatment, and who have not been isolated (Centres for Disease Control 2005, 2006; Curry International Tuberculosis Center 2007). All healthcare institutions need an infection-control programme in order to have rapid detection of TB, employ airborne precautions (such as opening of windows and wearing of N95 masks) and ensure treatment of people suspected of having TB or are diagnosed with TB (National Center for HIV/AIDS, Viral Hepatitis, STD and TB Prevention 2012). It is thus important to ensure that all hospital employees have adequate knowledge of all potential sources of infection, relevant control measures and practices to ensure that appropriate measures are implemented to reduce risk related to exposure to TB (National Center for HIV/AIDS, Viral Hepatitis, STD, and TB Prevention 2012).

Avoidable TB infections among employees can be prevented with good knowledge and practises related to infection control principles, underpinned by a good standard of hygiene. Of particular significance is having the personal discipline to undertake simple, repetitive tasks such as hand washing, frequently and thoroughly (National Center for HIV/AIDS, Viral Hepatitis, STD, and TB Prevention 2012). Furthermore, a good standard of facilities in order to clean, sterilise or disinfect possibly contaminated equipment, and the availability of signs indicating hygiene and cough etiquettes are essential to facilitate adherence to infection control principles (National Center for HIV/AIDS, Viral Hepatitis, STD, and TB Prevention 2012).

There is an urgent need to improve existing TB infection, prevention and control measures in specialised TB hospitals by providing training to healthcare workers and ancillary staff to develop good practices in applying organisational infection control policies and procedures (Grobler et al. 2016). This would help to promote a culture of good infection control practice among employees working in specialised TB hospitals (National Center for HIV/AIDS, Viral Hepatitis, STD, and TB Prevention, 2012). The currently poorly enforced infection prevention control policies, the over-crowded TB facilities with insufficient ventilation to allow for appropriate environmental infection control and the number of infectious TB patients all contribute to healthcare workers’ increased risk of exposure to TB in South African healthcare facilities in general, and Nelson Mandela Bay’s facilities in particular (Globler et al. 2016). It is therefore essential for hospital employees to receive ongoing education and training regarding occupational TB and infection control to ensure optimal infection control (World Health Organization 2004).

In South Africa, the health and safety of all employees at work is covered by the *Occupational Health and Safety Act*, No 85 of 1993 (Republic of South Africa 1993). There is also a national infection prevention and control policy for TB, multi-drug-resistant tuberculosis (MDR-TB) and extensive drug-resistant tuberculosis (XDR-TB), which acts as a guide for both management and employees to minimise the risk of TB transmission in healthcare facilities where there is increased risk of TB transmission (Department of Health 2014). Furthermore, screening and surveillance for TB among hospital employees is vital (Department of Health 2014). Interventions to improve efficiency, quality, safety and access to care have been introduced by the National Department of Health, the most significant to date being the framework for assessment of health establishments. Core standards were introduced to achieve this aim. Among the action areas covered by the safety domain is that of infection prevention and control, which intends to reduce occupational exposure to infections (including TB) through monitoring and management (Department of Health 2011). In spite of these interventions, the risks of contracting TB and rates of TB among hospital employees (also referred to as occupational TB) are poorly documented, especially in the Eastern Cape province. It is commonly known that knowledge is one of the potential promoting factors for good practices (Tada, Watanabe & Senpuku 2014). A number of studies have been conducted on the knowledge and practices of employees regarding infection control and occupational exposure to TB with various results. For example, a study conducted at Dr George Mukhari Academic Hospital in Rarankuwa revealed that the majority of participants had a good level of knowledge regarding TB-control measures, but more than 70% of participants did not comply with the correct practices in this regard (Mndzebele & Kandolo 2014). Farley et al. (2012) found that employees with a higher level of clinical training (e.g. medical doctors and allied health workers) are associated with a greater infection control knowledge and more appropriate attitudes and practices. Contrary, a study conducted in Botswana by Tlale et al. (2016) established that among categories with a reasonable level of clinical training, health education assistants and doctors had the same level of knowledge as other categories. This could be because of a number of factors including suboptimal quality or technique of training or insufficient knowledge retention (Tlale et al. 2016).

The hospitals in this study had active infection control programmes in place and were expected to provide the workers in these institutions with sessions where they are equipped with the relevant knowledge and practices to have information on infection control and occupational exposure to TB. However, the researcher observed discrepancies in terms of infection control practices. For example, employees only wore protective coats as protection against cold in winter but did not wear protective coats in summer. Because of lack of office space, there were no patient consultation rooms for part-time doctors which led to the nurse’s duty rooms being used for consulting patients. The same duty rooms that were used for patient consultations were used as dining rooms during tea and lunch breaks for staff. Even though there were hand-washing basins in all wards, employees seldom washed their hands. Furthermore, although patients are regarded as highly infectious during admission, neither of the employees who work in the admission desk wore a mask when admitting patients. General assistants, workshop employees and drivers also seldom wore masks even when working in the wards or while transporting patients. It was unclear whether workers had sufficient knowledge regarding these practices in order to prevent occupational exposure. Therefore, the researcher aimed to explore and describe the knowledge and practices of employees in three specialised TB hospitals in Nelson Mandela Bay in the Eastern Cape, South Africa, regarding occupational exposure to TB.

## Design and methods

A quantitative, descriptive and contextual study was conducted in 2016 at three specialised TB hospitals in Nelson Mandela Bay, in the Eastern Cape, South Africa.

At the time the study was conducted, the three hospitals A, B and C had approved bed capacities of 350 with a total of 100 employees (hospital A), 333 with a total of 90 employees (hospital B) and 186 with a total of 63 employees (hospital C). During the period the study was conducted, the total number of employees in all three hospitals was 253 of which 137 were nursing and clinical personnel, with the remaining 116 being non-clinical personnel, such as cleaning, maintenance and administration personnel. The hospitals are all specialised TB hospitals admitting patients diagnosed with TB responsive to all drug therapy, whereas hospital C also admits patients with drug-resistant TB types such as MDR-TB and XDR-TB. As the population size was small, a convenience sample of the entire population (*N* = 253) was used.

### Data collection tool

A self-administered questionnaire was used, which was adapted and piloted with permission from a study that was conducted by Bhebhe, Van Rooyen and Steinberg (2014). The questionnaire, which was written in English, contained 27 questions in three sections, namely, sections A, B and C. Section A included four items with demographic data, including age, gender, education level and clinical training, such as medical or nursing training. Section B (knowledge of TB) included four multiple-choice questions, seven true and false questions and three filter and follow-on questions regarding an infection control policy. Section C included the following questions regarding practices of employees regarding infection control: four filter questions regarding personal practices, two questions regarding protective clothing, such as masks and hand washing, and three filter questions regarding institutional practices – the availability of consultation rooms, dining hall and patient transportation. Questions were asked regarding standard infection control precautions as well as specific precautions for TB.

To measure participants’ knowledge and practices on a quantitative scale, various techniques were used to convert the responses to questionnaire items to scores in the range 0 (all incorrect or inappropriate responses) to 10 (all correct or appropriate responses). The *knowledge* score was the average of the scores for each of the six sections on knowledge. The *personal practices* and *institutional practices* scores were related to the questionnaire items on personal and institutional practices, respectively.

Based on the guidelines of infection control practice, a judgement was made about whether an answer reflected sound, fair or poor knowledge and good, fair or bad practice. With the assistance of a senior statistician, it was determined that a high score was regarded as between 6.01 and 10.00, an average score from 4.00 to 6.00 and a low score from 0.00 to 3.99. These scores were calculated as follows: quartiles 1 and 3 were used to separate respondents into three groups: lower group: score less than quartile 1; middle or average group: score between (inclusive) quartiles 1 and 3; and higher group: score greater than quartile 3. This technique ensures that approximately 25% of the respondents are in the lower group, 50% in the middle group and 25% in the higher group.

Validity of the questionnaire was ensured in the current study by conducting a literature review and seeking the advice of experts such as the researcher’s supervisors and statistician to ensure that the questionnaire adequately covered the research question. Reliability was ensured by conducting a pilot study.

### Data collection process

The data collection using the self-administered questionnaire was conducted by the first author in December 2016. Appointments with the hospitals were made prior to the data collection date. On the data collection date, all participants were gathered in one venue and were informed about the project, and the research process was explained to them. Data collection for the three hospitals took 3 days (1 day per hospital). The data collection was conducted on site, using the communal hall in each hospital, after obtaining signed, informed consent from each participant. The participants were requested to answer the questionnaires independently. The questionnaire took approximately 5–10 min to complete, and the questionnaires were collected on the same day. Questions were asked in English as this was the language the participants were proficient in.

### Data analysis

The data were captured and analysed using descriptive and inferential statistics. A Microsoft Excel spreadsheet was prepared based on the pre-coding performed in the data-gathering instrument. Descriptive statistics, including frequency distributions, means and standard deviations, were used to summarise and describe the demographic profile of the sample and the knowledge scores obtained by the respondents. The following inferential statistics were used to investigate the relationships among variables: chi-square test, Pearson product moment correlation and analysis of variance (ANOVA).

### Pilot study

To test the questionnaire, a pilot study was conducted over a 1-week period in November 2016, prior to the data collection. The pilot study utilised five employees from different areas of work, namely, administration, admissions, clinical, general assistant and nursing in one of the specialised TB hospitals in the Nelson Mandela Bay. After the pilot test, there were minor amendments to the questionnaire including adding extra options such as ‘I don’t work with TB patients’ and ‘I don’t know’ as these options were absent in the original questionnaire. The pilot study results were not included in the data analysis of the actual study.

### Ethical considerations

Ethics approval was obtained from the Ethics Board of the Faculty Postgraduate Studies Committee (FPGSC) at Nelson Mandela University (ethics number H16-HEA-NUR-025) as well as from Eastern Cape Department of Health (ethics number EC_2016RP28_145). Permission was obtained from the Chief Executive Officers who acted as gatekeepers of the three TB hospitals included in the study. No names or identifiers were recorded in the questionnaires to ensure the anonymity and confidentiality of the employees. Individual written informed consent was obtained from each participant. The participants were informed that their participation was voluntary and that they had the right to withdraw at any time. A copy of the questionnaire was kept in a locked place and accessed by the second author only.

## Results

The overall response rate was high, with 181 out of a potential 253 employees agreeing to willingly participate in the study. The sample thus represents 72% of all workers employed by the three specialised TB hospitals in Nelson Mandela Bay. The results per section of the questionnaire will now be outlined.

### Demographic data (Section A)

The demographic profile of the sample is reflected in Table 1.

**TABLE 1:** Demographic profile of participants.

Variable	*n*	*%*
**Age in years,**		
18–25	11	6
26–35	35	19
36–45	62	34
46–55	42	23
> 55	30	17
Not specified	1	1
**Female**	129	71
**Highest level of education**		
Grade 1–7	1	1
Grade 8–12	113	62
Degree or diploma	49	27
Other	5	3
Not specified	13	7
**Clinical training received**		
Clinical and nursing	67	37
Non-clinical	108	60
Not specified	6	3

It was concluded from the demographic data that approximately a third (34%) of the participants were between the ages of 36 and 45 years and the majority were women (71%). Most of the participants (62%) indicated high school as their highest level of education and less than a third of the participants (27%) had a degree or diploma as the highest level of education. The majority of participants (60%) had not received any clinical training.

### Knowledge of employees regarding tuberculosis and infection control (Section B)

Section B of the questionnaire had various questions to determine the knowledge of employees regarding TB and infection control, standard infection control precautions such as washing hands, as well as precautions for TB. The extent to which participants gave the correct responses to these questions is summarised in Table 2.

**TABLE 2:** Knowledge of tuberculosis (*n* = 181).

Items	Correct response	Percentage correct	
		*n*	*%*
**B1 How is TB transmitted from one person to another?**			
Waterborne	No	177	98
Airborne	Yes	140	77
Direct contact	No	150	83
Sexual contact	No	179	99
Blood contact	No	178	98
All of the above	No	168	93
I do not know	No	178	98
**B2 What are the symptoms of TB?**			
Cough more than or equal to 2 weeks	Yes	164	91
Blood in the stools	No	172	95
Loss of weight	Yes	149	82
Oral thrush	No	176	97
Fever	Yes	118	65
Chronic diarrhoea	No	167	92
Night sweats	Yes	140	77
I do not know	No	179	99
**B3 How is (extra)pulmonary TB diagnosed?**			
Stool culture	No	177	98
Sputum smear	Yes	165	91
Pleural fluid aspirate	Yes	22	12
Cerebrospinal fluid analysis	No	170	94
All of the above	No	180	99
I do not know	No	177	98
**B4 Which type of TB spreads from one person to another**			
TB meningitis	No	162	90
TB pleuritis	No	170	94
TB pericarditis	No	176	97
TB lymphadenitis	No	181	100
TB spine	No	181	100
TB peritonitis	No	179	99
Pulmonary TB	Yes	146	81
I do not know	No	164	91
**B5 True or false statements**			
TB is treated for at least 6 months	True	168	93
TB is preventable	True	159	88
HIV makes a person more vulnerable to TB	True	153	85
Washing hands with soap reduces the spread of infection	True	159	88
A person needs to wear a protective coat and gloves before entering the isolation ward or area	True	160	88
A person needs to take off the gown and gloves before leaving the work area	True	168	93
A person needs to wash hands before leaving the isolation ward or the work area	True	169	93

Note: Items with less than 90% correct responses are in bold text.

TB, tuberculosis; HIV, human immunodeficiency virus.

As reported in Table 2, more than 90% of the participants gave the correct response for most of the questionnaire items in Section B – Knowledge. The items with less than 90% correct responses are in bold text in Table 2.

### Knowledge regarding infection control policy

When asked about whether there is an infection control policy in the hospital, the majority of participants (78%, *n* = 141) knew about its availability; however, only approximately half of the participants (49%, *n* = 89) reported to have actually read the document. Slightly more than half of the participants (58%, *n* = 105) indicated to have received training on the infection control policy.

### Practices of employees regarding infection control (Section C)

The questionnaire items in Section C relating to practices were divided into personal and institutional practices. The extent to which participants gave the correct responses to these questions are summarised in Table 3.

**TABLE 3:** Practices of participants – correct responses (*n* = 181).

Items	*n*	*%*
**Personal practice**		
Windows are always kept open for ventilation and sunlight	172	96
Hand washing is always performed after taking care of TB patients	137	94
Masks are used at all times when in the hospital premises	**38**	**22**
Protective gowns are always worn when attending to TB patients	**32**	**19**
**Institutional practice**		
Availability of staff or employee dining hall	**121**	**69**
Availability of consultation rooms in all the wards	**97**	**57**
Availability of isolation glass (in patient vehicle) between the driver and the patients during the journey	**37**	**21**

Note: Items with less than 90% correct responses are in bold text.

TB, tuberculosis.

According to Table 3, the only items with more than 90% correct responses were regarding opening of windows (96%) and hand washing after taking care of TB patients (94%). These responses indicated the participants’ knowledge of best practice in the control of TB in the ward situation, that of essential cross-ventilation. The lowest correct percentages were observed for wearing protective gowns (19%), masks (22%) and the availability of isolation glass in patient vehicles (21%). It appears as though the wearing of masks and protective clothing is not a regular practice. Staff noted the lack of isolation glass in the patient vehicle but it was not noted if they understood the necessity for the use thereof, that of adequate practice in the prevention of transmission of TB. Staff noted the absence of a dining hall and concurred that most staff eat lunch in their offices, or in the vicinity of the wards, which means limited time away from infected patients.

**TABLE 4:** Descriptive statistics – Knowledge and practice scores.

Items	Mean	Standard deviation	Frequency distribution										
			Low (0.00–3.99)		Average (4.00–6.00)		High (6.01–10.00)		Total	
			*n*	*%*	*n*	*%*	*n*	*%*	*n*	*%*
B1 TB transmission	7.27	4.20	41	23	17	9	123	68	181	100
B2 TB symptoms	6.74	2.90	44	24	13	7	124	69	181	100
B3 TB diagnostic tool	3.61	1.89	164	84	0	0	17	10	181	100
B4 Type of TB that spreads	7.82	3.99	35	19	9	5	137	76	181	100
B5 General knowledge about TB and infection control	9.23	1.57	4	3	3	2	168	96	175	100
B6 Availability of infection control policy	5.42	3.03	77	48	0	0	83	52	160	100
Knowledge (average of B1–B6 scores)	6.65	1.69	12	7	33	18	136	75	181	100
Practice personal	6.12	2.03	38	21	23	13	120	66	181	100
Practice institutional	6.15	2.02	24	13	66	36	91	51	181	100

TB, tuberculosis.

### Knowledge and practice scores

Descriptive statistics for the various summated scores that were calculated to measure participants’ knowledge and practices are reported in Table 4. As described in the Methods section, all the scores are in the range from 0 to 10, with scores from 6.01 to 10.00 regarded as high, from 4.00 to 6.00 as average and from 0.00 to 3.99 as low.

According to Table 4, mean values that can be described as good (*M* > 6.00) were observed for most of the knowledge and practice scores, the exceptions being: B3 TB diagnostic tool (*M* = 3.61, 10% high) and B6 Availability of infection control policy (*M* = 5.42, 52% High).

The majority of the participants displayed a good knowledge of TB (*M* = 6.65, 75% high) and had good scores for practice personal (*M* = 6.12, 66% high) and practice institutional (*M* = 6.15, 51% high).

### Relationship between knowledge and personal practices

The relationship between participants’ knowledge and their personal practices was found to be non-significant (*r* = 0.033, *p* = 0.659; Chi² [df = 4, *n* = 181] = 6.83; *p* = 0.145). The relationship between knowledge and institutional practices was not investigated, given that institutions’ practices are not related to their employees’ personal characteristics.

### Relationships between socio-demographic characteristics and knowledge and practice

The results of the ANOVA that was conducted to investigate the relationships between the participants’ socio-demographic characteristics and their TB knowledge and practice are summarised in Table 5.

**TABLE 5:** Analysis of variance results – knowledge and practice personal by demographic variables (*n* = 175).

Items	Knowledge			Personal practice		
Effect	*F*	df	*p*	*F*	df	*p*
Age	2.78	3; 171	0.0430	0.29	3; 171	0.831
Gender	2.19	1; 171	0.1400	2.23	1; 171	0.138
Education	19.38	1; 171	< 0.0005	0.21	1; 171	0.648

df, degrees of freedom, *F, F* statistic.

It is concluded from Table 5 that the only statistically significant relationships were those between age and knowledge and between education and knowledge. Post-hoc tests for these relationships revealed the following:

- Age and knowledge: The 36–45 years age group (*n* = 61, *M* = 6.31, *s* = 1.68) had a significantly (Scheffé’s *p* = 0.041, Cohen’s *d* = 0.55) lower level of knowledge regarding average than the 46–55 years age group (*n* = 41, *M* = 7.25, *s* = 1.70). No significant differences were observed for the 18–35 years age group (*n* = 45, *M* = 6.51, *s* = 1.57) nor for the over 55 years age group (*n* = 30, *M* = 6.69, *s* = 1.80).
- Education and knowledge: The group with a degree or diploma (*n* = 49, *M* = 7.48, *s* = 1.40) had a significantly (Cohen’s *d* = 0.72) higher level of knowledge regarding average than those with neither a degree nor diploma (*n* = 128, *M* = 6.32, *s* = 1.70).

## Discussion

Participants in this study generally displayed high knowledge and practice scores regarding TB and infection control, although scores for knowledge were generally higher than those for practices. Therefore, it can be concluded that participants in this study were knowledgeable about TB and infection control; however, they did not always practise what they knew. This is similar to the findings of a study conducted in Rarankuwa, which revealed that 93% of the participants had a good level of knowledge regarding TB-control measures, but the majority (about 70%) of participants did not comply with the correct practices in this regard (Mndzebele & Kandolo 2014). Furthermore, a similar study conducted regarding knowledge, attitudes and practices of general assistants towards infection control at a hospital in Lesotho displayed both moderate knowledge and practices of infection control (Peta 2014).

Most employees in this study were aware of the availability of an infection control policy, but this policy was kept in the infection controller’s office, and therefore, employees did not always read the policy. Similar results to the current study were displayed in a qualitative study conducted regarding the TB infection prevention and control experiences of South African nurses. The results revealed knowledge about the availability of a TB infection policy at the hospital; however, most participants were unaware of its content (Sissolak, Marais & Mehta 2011). In the aforementioned study, a hospital TB policy existed but seemingly had not been made known to all ward nurses (Sissolak et al. 2011).

Items on knowledge that specifically obtained lower scores included transmission of TB, symptoms of TB and diagnostic tools and the type of TB that spreads. Airborne and direct contacts as modes of TB transmission were not answered correctly by participants. Participants failed to identify loss of weight, fever and night sweats as symptoms of TB. Furthermore, participants failed to identify pleural fluid aspirate analysis as a TB diagnostic tool. Pulmonary TB as the type of TB that spreads from person to person was also not correctly answered by all participants. Some of the true or false statements were also incorrectly answered such as the statements on HIV making a person more vulnerable to TB, as well as TB being a preventable disease. A survey conducted regarding knowledge, attitudes and practices on TB among healthcare workers in Kingston & St. Andrews, Jamaica, yielded slightly different results (White 2011). Most participants in the survey managed to identsify cough for more than 2 weeks, coughing of blood, weight loss, night sweats as well as fever as symptoms of TB (White 2011). Regarding TB diagnostic tools, most participants in the survey failed to identify sputum smear culture as a primary diagnostic tool (White 2011).

Practices that were particularly not adhered to and yielded a low score in this study included wearing personal protective equipment such as gowns and masks, which was also found in similar studies (Sissolak et al. 2011; White 2011). Another practice that was not well adhered to is the availability of isolation glass in patient vehicles. This should be adhered to as there is a general finding that using public transport independently is associated with a high risk of contracting active TB (Feske et al. 2011; Furukawa et al. 2014).

The current study indicated a strong association between knowledge and age (specifically the 36–44 age group) and knowledge and education level (specifically diploma and degree). Previous studies have shown that knowledge and practices can be improved with provision of appropriate supplies and strengthening training and supervision (Peta 2014). More than half of the participants did not have any clinical training, and did not possess a degree or diploma. It is therefore recommended that training should be provided to non-nursing or non-clinical personnel, taking into consideration their scope of work, level of education, level of clinical training and specific risks attached to their work environment. Furthermore, training material matching the tasks and education level of non-clinical employees and facilitation of opportunities to learn and read about infection control needs to be designed. Specific attention should be given to TB transmission, symptoms, diagnostic tools as well as the type that spreads TB in the in-service training. Furthermore, information and demonstrations should be given on the wearing of protective gear in order to reduce the occupational exposure to TB. Finally, specific attention should be given to the quality or technique of training and ways in which sufficient knowledge retention can be established as Tlale et al. (2016) observed the same level of knowledge among categories with a reasonable level of clinical training such as health professionals as compared to other categories.

In addition, management in specialised TB hospitals must ensure the availability of an infection control policy to all employees. The implementation of the infection control policy should be monitored by management. Furthermore, management should ensure the availability of masks as well as placing isolation glass in patient transport vehicles at all times, as these were the practices that were scored the lowest by the participants in this study.

Several limitations were observed. Questions, such as the Xpert as an option for the question how (extra)pulmonary TB is diagnosed as well as questions about respiratory precautions in the questionnaire, could have been included in the questionnaire. However, the questionnaire was developed for participants with both clinical and non-clinical backgrounds. An item establishing whether participants attended any information-sharing sessions on the knowledge and the expected practices was not included in the questionnaire, but would have added value. It is therefore recommended that the questionnaire should be further revised and tested.

This study was the first of its kind to explore and describe knowledge and practices regarding occupational exposure to TB in specialised TB hospitals in the Eastern Cape. Considering the limited research performed on occupational exposure to TB, especially in the Eastern Cape, further explorative studies could be conducted as to why certain knowledge and practices scored lower. Research in terms of impact studies can be performed to determine the effectiveness of the infection control training offered by the hospitals, particularly related to implementation of the knowledge. In addition, the current study can be replicated in all specialised TB facilities in the Eastern Cape, as this will allow for further validation of the questionnaire as well as be of benefit in determining the knowledge and practices of a wider population of employees in such facilities.

## Conclusion

In conclusion, the knowledge of employees regarding occupational exposure in specialised TB hospitals in the Nelson Mandela Bay scored generally high. A strong correlation was found between knowledge and age as well as knowledge and education level. Further opportunities for education, practice and research to explore and investigate occupational exposure to TB were provided.
